# Stroke survivor perceptions of using an exoskeleton during acute gait rehabilitation

**DOI:** 10.1038/s41598-022-18188-7

**Published:** 2022-08-19

**Authors:** Caitlin McDonald, Caitriona Fingleton, Sean Murphy, Olive Lennon

**Affiliations:** 1 School of Public Health, Physiotherapy and Sports Science, University College Dublin, Dublin, Ireland; 2 Department of Physiotherapy, Mater Misericordiae University Hospital, Dublin, Ireland; 3 Department of Geriatric and Stroke Medicine, Mater Misericordiae University Hospital, Dublin, Ireland; 4 School of Medicine, Royal College of Surgeons, Dublin, Ireland; 5 School of Medicine, University College Dublin, Dublin, Ireland

**Keywords:** Health care, Engineering

## Abstract

Robotic-assisted gait training (RAGT) devices allow intensive high repetition of the gait cycle in individuals with locomotor disability, with reduced therapist effort. In addition to usual rehabilitation, RAGT post-stroke improves the likelihood of regaining independent walking, with maximum efficacy identified in the acute and subacute phases of stroke. This study explores the usability and acceptance of RAGT among persons with stroke in an acute hospital setting and examines users’ perceptions of two different modes of robotic assistance provided during rehabilitation. A mixed-methods approach comprised semi-structed interviews of end-user perspectives of RAGT in an acute hospital setting following stroke and two 10-point Likert scales rating how comfortable and how natural robotic gait felt using different assistance modes. Content analysis of qualitative data was undertaken with results synthesised by common meaning units. Quantitative data were reported using summary statistics, with Spearmann’s correlation co-efficient examining the relationship between Likert scale ratings and measures of participants’ stroke related disability. Ten individuals (6 men; 4 women; mean age of 64.5. ± 13 years) were recruited in an acute hospital setting following admission with a stroke diagnosis. Content analysis of interview transcripts identified discussion units centring around positive aspects of how helpful the device was, negative aspects related to set-up time, weight of the device and multiple instructions delivered during use. Initially participants identified that the device could look intimidating, and they feared falling in the device but they subsequently identified the correct mindset for using the device is to trust the technology and not be afraid. Mean ratings for device comfort (7.94 ± 1.4) and how natural walking felt (7.05 ± 1.9) were favourable. Interestingly, a strong relationship was identified, whereby the higher the level of disability, the more natural participants rated walking in the device during maximal assistance mode (rho = 0.62; *p* = 0.138). This study suggests individuals in the early phases of stroke perceive RAGT to be acceptable and helpful in the main, with some associated negative aspects. Walking in the device was rated as comfortable and natural. Those with greater disability rated the assisted walking as more natural.

## Introduction

Stroke is a leading cause of mortality and morbidity worldwide. In 2019, over 6.5 million deaths and 143 million disability-adjusted life-years (DALYs) resulted from stroke globally^1^. Hemiplegia is one of the most common impairments after stroke and significantly reduces locomotor performance^2,3^. Although the majority of stroke survivors achieve independent gait, many do not reach a walking level that enables them to perform all activities of daily living^4^. For example, only 7% of patients discharged from rehabilitation following stroke reported meeting the criteria for community walking^5^. At six months post-stroke 11% of survivors are unable to ambulate, with only 14% recovering independent gait with no walking aids^6^. Individuals following stroke commonly demonstrate an altered gait pattern^7^, in compensation for impairments such as neuromuscular weakness, altered muscle tone, poor motor control, and soft tissue contractures. This results in increased energy expenditure during overground walking when compared to healthy individuals^8^. Gait recovery is a major rehabilitation objective post-stroke^9^, with stroke survivors and other relevant stakeholders identifying gait rehabilitation and specifically electromechanically assisted gait training as a main priority for future research^10^. Effective rehabilitation for this population requires high intensity, repetitive, and task-specific training^11^, with intensity of practice strongly associated with functional gait outcomes^12^.

Exoskeleton or robotic gait devices enable people to walk with electromechanical assistance to achieve a more normal gait pattern and can allow intensive high repetition of the gait cycle with reduced therapist involvement^13^. The addition of robotic-assisted gait training (RAGT) to usual post-stroke rehabilitation has been shown to improve the likelihood of regaining independent walking following a stroke^14^. While combining exoskeleton-based gait training with conventional physiotherapy can promote improved mobility and favourable neuroplastic changes in patients with chronic stroke^15^, it is in the first three months after stroke (acute and subacute stages) when robotic gait training has shown most efficacy by meta-analysis^14^. This is in keeping with data that supports the majority of positive neuroplastic change as occurring in the subacute stage^16^, during a time when the brain’s response to injury is heightened and rehabilitation may be more effective^17^. The best achievable neurological outcome is usually reached (in 80% of all patients) within five weeks, with 95% reaching their best outcome of functional ability at three months after stroke onset^18,19^.

Despite evidence of efficacy for RAGT in conjunction with routine rehabilitation in the earlier phases after stroke in gait restoration, limited data provide insights into stroke survivors, as the primary end-users, perceptions of these devices in the acute stage.

Stakeholders’ perceptions of robotics in motor rehabilitation are reported by qualitative meta-synthesis, to be generally acceptable, useful and beneficial (physically, psychologically, and socially) in rehabilitation^20^. However, this review included only one qualitative study specifically focussed on gait rehabilitation after stroke^21^, including only individuals in the chronic phase of stroke. Since this review was conducted, two further qualitative studies of RAGT in the chronic stage of stroke (> 12 weeks)^22^ and in the subacute phase following transfer to a rehabilitation centre^23^ were identified. In these later stages, exoskeleton interventions were generally well-received by physical therapists and stroke survivors with expressed excitement and hopefulness about exoskeleton technology. However, disappointment was registered by some individuals where the technology did not yet meet their expectations. A more adaptive device that augmented their natural gait as opposed to one that imposed a pre-set gait pattern was desired. Future pairing of questionnaires with interview-based data to better quantify acceptance and explore potential relationships between demographic or functional characteristics and user perspectives was recommended^23^. Recent quantitative studies^24,25^ have further evaluated the acceptance of RAGT in individuals undergoing rehabilitation in the subacute phase of stroke (up to 6 months post event) during a maximal assistance training programme using the Technology Acceptance Model questionnaire^24^ and a similar questionnaire where comfort, presence of pain, presence of fatigue, enjoyment, advantages, desire to continue, and likelihood to recommend to someone were rated^25^. Results identified RAGT in maximal assistance mode was positively perceived as useful, considered comfortable and enjoyable, was moderately painful and strenuous and the majority of participants would recommend it and would like further RAGT^24,25^.

This mixed-methods study first gathers and explores qualitative data relating to end-user perspectives of using an exoskeleton gait training device in the acute hospital sector after stroke, thus contributing to the current knowledge gap around usability and acceptance of robotic-assisted gait training in the earlier phase of stroke and during a window of potentially enhanced neuroplasticity. In addition, it gathers preliminary quantitative data from Likert scales rating how comfortable and how natural walking feels in the device using different assistance modes and examines the relationship between these scales and participants’ ambulatory capacity and stroke related disability.

## Materials and methods

### Ethics approval and consent to participate

This study was approved by the hospital’s (MMUH) Research Ethics Committee reference 1/378/2052 and written informed consent was obtained from all participants. All methods were performed in accordance with relevant guidelines and regulations.

### Consent for publication

Written informed consent was obtained from all participants.

### Participant recruitment

Participants were volunteers by self-selection, recruited from an acute hospital setting. Participants were informed of the study by their treating therapist and an information leaflet was provided.

Inclusion criteria required participants to have had a recent hospital admission with stroke, be over 18 years of age, and be medically stable at the time of the research participation. Participants had to measure between 1.58 and 1.88 m in height with a maximum hip width of 0.46 m and a maximum weight of 100 kg, to satisfy the dimensions of the exoskeleton. Exclusion criteria included significant cognitive impairment, other neurological conditions or co-existing conditions that limited device use, severe spasticity (modified Ashworth Scale = 4)^26^ in one or more of the opposing muscle groups at the ankle, knee or hip joints, and acute medical illness. Potential participants with aphasia were not excluded unless the impairment limited their capacity to provide informed consent or follow instruction.

### Data collection

Following informed consent, participants attended one familiarisation day, where demographic and clinical data were recorded from the medical chart, anthropometric measurements were taken and the exoskeleton (EKSO GT, California USA) dimensions were adjusted accordingly for individual participants. Participants were evaluated by their treating therapist at the MMUH stroke unit and assigned a disability level using the modified Rankin Scale (mRS) and Functional Ambulation Category (FAC) prior to admittance to the study. This information was recorded by the research team from the medical charts. Participants completed an introductory RAGT session (lasting approximately 45 min), and one follow up gait training session during which data collection using Likert rating scales occurred. Qualitative interviews were then conducted post training, on the same day, where possible, or within two days of training otherwise. During the introductory session step length, step height, and lateral weight transfer targets were individually customised to each participant by a physiotherapist certified in the use of the device. Participants completed repeated 10 m walks, with rests allowed as required. At the next training session, participants completed gait training using both maximum assistance (MA) and adaptive assistance (AA) modes. The order of use of each mode was determined by a randomised schedule prepared in advance. In both modes, the exoskeleton was programmed to the “ProStep” mode, in which forward and lateral movement of the users’ bodyweight triggers the next step^27^. In MA mode the initiation of movement requires only the ability to maintain balance and transfer bodyweight and the device moves the lower limb in the predefined stepping pattern. In AA mode, the participant provides the limb movement where possible, and the device completes the stepping activity where it fails to meet the predefined trajectory parameters.

Interviews were conducted by an experienced qualitative interviewer (OL) after the robotic gait training sessions. A structured but flexible question schedule was used that allowed participants to elaborate freely on their own experiences and perceptions. The semi-structured interviews were audio-recorded, transcribed verbatim and the recordings were subsequently deleted. Key questions asked during interviews are available in Table 1.

**Table 1:** Interview questions.

*What was your first impression of the device?*
*What did you like about using the device, if anything?*
*What, if anything, do you think using the device could help with during your recovery?*
*What advice would you give to other people who have had a stroke, if they were going to use the device?*
*What ideas do you have for improving the device?*
*What advice would you give to physiotherapists using the device to help make it a good experience for patients?*

Following each training session in maximum and adaptive modes, participants were asked to provide ratings for two questions using 10-point Likert scales. Firstly, ‘rate how comfortable you felt during that walk’, with 1 being very uncomfortable and 10 being very comfortable. Secondly, ‘rate how natural that walk felt to you’, with 1 being not natural at all and 10 being very natural. Ratings were recorded for both MA and AA walking modes.

### Data analysis

Content analysis was employed to analyse qualitative interview data and results were synthesised by reporting common meaning units identified in data. Coding was completed by labelling data segments keeping to the participants’ own wording as much as possible. As data analysis progressed, data were assembled into categories by identifying common issues, drawing comparisons between participants as well as comparing data from the same participants. To ensure the reliability of the data analysis, two members of the research team (OL) (CMD) reviewed the data and developed the main categories by consensus. This method was deemed suitable as we were conducting exploratory work in an area where not much is known^28^.

Summary statistics were used to describe the characteristics of the study participants and to quantify how comfortable and how natural walking in a robotic-assisted gait device felt during the acute and subacute phases of stroke, as rated using the 10-point Likert scales. Spearmann’s correlation coefficient explored the relationship between walking capability (using the FAC), stroke related disability (using the mRS) and ratings for perceptions of device comfort and of how natural walking felt. As guided by Cohen (^29^, pp 79–81) a rho of 0.1 to 0.29 was considered indicative of a small relationship, 0.3 to 0.49 a medium relationship, and 0.5 to 1.0 as a large relationship between variables^29^.

## Results

Ten clinically stable individuals (6 men; 4 women) were recruited in an acute hospital setting (Mater Misericordiae University Hospital (MMUH), Dublin) following admission with a stroke diagnosis. All ten underwent RAGT following familiarisation, eight of whom consented to the semi-structured interview and nine of whom provided data using the Likert rating scales.

Participant demographics and clinical characteristics are summarised in Table 2. A detailed profile of each participant is provided as a supplementary file (Table 1). This includes participants’ weight, height, stroke details, mobility status, use of aids and appliances, speech and language difficulties, cognition and consciousness screening tools recorded from participants’ medical records and interpreted as per user guidelines^30–32^.

**Table 2:** Participant demographics and clinical characteristics.

	N = 10
	Mean (SD)
Age at time of data collection (years)	64.5 ± 12.99
Time since stroke (days)	35 ± 21.9
MRS pre stroke	0.6 ± 1.26
MRS post stroke	4.1 ± 0.99
FAC pre stroke	5 ± 0
FAC post stroke	1.44 ± 1.19

	N (%)
Male sex	6 (60%)
Stroke laterality-left	6 (60%)
Stroke type-ischemic	9 (90%)

Participants had a mean age of 64.5. ± 12.99 years and were on average 38.9 days (range 14–79) post-stroke when recruited to the study. Eight participants had strokes of ischemic origin, one of haemorrhagic origin, and one with mixed aetiology. All were first episode strokes. Eight participants (80%) had post-stroke FAC scores from 0 to 2 indicating they could not ambulate independently, the remainder (N = 2) could mobilise without physical assistance but required close supervision due to balance problems^33^. Three participants, detailed in the supplementary file (Table 1) were documented as having tonal changes in lower limb muscles of the stroke affected limb. These were measured on the modified Ashworth Scale as 1/4 in the knee flexors in one participant, 2/4 in the knee flexors of a second participant and as 1/4 in the plantar flexor muscles in third participant. One participant, who was not a native English speaker, required a translator for the interview to be conducted and the Likert scales were verbally translated. One participant had expressive aphasia and a closed question interview style was adopted to aid communication during the qualitative interview.

### Qualitative interviews

Content analysis of the interview transcriptions identified primary categories of discussion items that centred around positive aspects of the device, negative aspects, initial concerns relating to the device, the correct mindset for using the device and advice for therapists using the device in an acute rehabilitation setting. Interview data, categories and codes can be found in Table 3.

**Table 3:** Interview data.

Categories	Common issues reported in data (codes)	Frequency of codes*	Example of quotes
Positive aspects of robotic gait device	Belief device is a beneficial addition to rehabilitation	8	“Deep down I do say it will do something good.” “It will probably help me.”
	Intimidating initially but subsequently positive	3	“It's quite intimidating looking… 'cause you don't think you're going to be able to move in it…And you realize that what the weight is, it's not as bad as “It was crazy!… I enjoyed it”
	Feeling comfortable using the device	4	“I had no fear of it, even though they were talking about this that and the other, but I didn't feel any fear.” “None of it was ever a bad experience”
Negative aspects of robotic gait device	Device fitting time	2	“Too many sensors… too time to, to, yeah, to put the device on your body. And bells and stirrups and, yeah, require more shorter time”
	Weight of device	3	“Just the weight… 'cause you really feel like you're- you're really are putting the effort in” “The weight was so heavy.”
	Degree of verbal instruction required from physiotherapists	1	“… I think sometimes the instruction's hard to understand. Like I'm saying move to the right, move to the left, that kind of thing sometimes hard. Maybe less instruction.”
	Discomfort	1	“I find it quite uncomfortable.”
	Fear of falling in familiarisation phase	1	“Feelings of fall, you fall, fall on the floor, you know. In the beginning”
Advice for future patients using the device	Have an open mind	1	“Go in with an open mind.”
	Not to be afraid	3	“Not to be afraid of it.”
	Trust the device	1	“Just go with it. Trusting. They're not going to fall over. The device will hold you up.”
	Relax	2	“Not to be in a rush like I was… Take it easy.” “Try and relax.”
Advice for future physiotherapists using the device	Use non-clinical language	1	“Explain in layman's terms.”
	Provide less instruction	1	“Maybe less instruction.”
	Experience using device personally	1	“For them to use it first…. To know what it's like. To feel how the patient will feel.”
	Continue as currently	3	“I don’t think there’s anything better they can do.”
			“You couldn't have done anything different.” “They're doing everything they can.”

*Frequency of codes indicates the number of times the code was mentioned in the transcripts.

### Narrative summary of qualitative data

The majority of participants discussed their engagement with the exoskeleton gait device as a positive experience in their rehabilitation. *“It's a good beginning”* (Participant #04) Participants described the device as increasing their confidence and improving their gait. *“It helps you concentrate more on your steps. It's very beneficial. It gives you confidence.”* (Participant #10).

The utility of the device during acute stroke rehabilitation for those unable to walk was identified. *“I think it's very good because I wasn't able to move around. All I wanted to do was stay in the bed at the hospital. You know? And now I made steps with the help*.” (Participant #01) There was one exception where a person who could mobilise independently but with balance deficits commented *“I don't think I'd need to* [use to help in stroke recovery]” (Participant #08) indicating that it may not be universally beneficial for all stroke survivors.

A number of negative aspects were identified. Approximately 30 min was required for each participant to take measurements, fit and set up the device before initial use with a number of participants commenting *“you spend, (many) more time on preparing than going go walking*” (Participant #04). The weight of the device was further considered by some as a negative feature, *“The weight was so heavy. I find it quite uncomfortable*” (Participant #08) along with the degree of verbal instructions provided by physiotherapists during robotic gait.

*“… I think sometimes the instructions [are] hard to understand. Like I'm saying move to the right, move to the left, that kind of thing sometimes [was] hard. Maybe less instruction.”* (Participant #08).

A number of participants identified initial concerns that warrant consideration clinically. These included that the device could be intimidating in the initial stages and that users worried about its restrictions. *“It's quite intimidating looking. ‘Cause you don't think you're going to be able to move in it. Because it's the big steel frame around you. Yeah, but once you have started… And you realize that… what the weight is, it's not as bad as you think it…. you think it's going to restrict your movements also. But it doesn't.”* (Participant #10).

For some, the device was further associated with a fear of falling in the familiarisation phase. *“Good device, but very, feelings of fall, [that you might] fall, fall on the floor, you know. In the beginning.”* (Participant #04) Participants identified that nervousness and fear could be an issue or barrier to engagement with the exoskeleton gait training device and advised future users *“Don't be as nervous as myself*.” (Participant #02) and *“Go in with an open mind. Don't be afraid of it.”* (Participant #09) *The device will hold you up.”* (Participant #10).

Participants identified that it would be beneficial for users of the device to have a clear idea of what was going to happen at every stage of use, and that advice should be given by the treating therapist in concise, accessible, and non-clinical language. *“It could be explained to people in layman's terms…. What it would do”* (Participant #09) and *“For them* (physiotherapists) *to use it first. To get into it… To know what it's like. To feel how the patient will feel.”* (Participant #10).

### Likert scale results

Summary results of ratings of comfort when using the device and how natural walking in the device felt for each exoskeleton mode are depicted in Table 4. Overall ratings of device comfort (mean rating 7.95; sd 1.4) and how natural walking felt (mean rating 7.05; sd 1.9) were favourable, with only one participant giving a rating below 5 on any scale (the natural feel of the device). This participant was able to ambulate independently with supervision at the time of data collection. Results indicate that participants rated walking in the device as more comfortable when walking in MA mode (mean Likert score 8.4) when compared to AA mode (mean Likert score 7.6). However, overall participants rated their gait as more natural when in AA mode (mean Likert score 7.3) when compared to MA mode (mean Likert score 6.8).

**Table 4:** Likert scale results.

Question	Mode	Mean ± SD
How comfortable?	Maximum assistance	8.38 ± 1.06
How comfortable?	Adaptive assistance	7.56 ± 1.59
How natural?	Maximum assistance	6.75 ± 1.75
How natural?	Adaptive assistance	7.33 ± 2.18

Likert scale where 1 = very uncomfortable and 10 = very comfortable or 1 = not natural at all and 10 = very natural.

The relationship between both participants’ stroke-related ambulatory category as measured by the FAC and disability as measured by the mRS and the Likert rating scales were next explored using Spearmann’s correlation coefficient. Of note, a strong and positive relationship (rho  = 0.6; *p* = 0.14) between the mRS and how natural walking in the device was rated in MA mode was identified, whereby the higher their level of disability, the more natural participants rated walking in the device in MA mode. A moderate and negative relationship was observed between FAC and how natural walking in the device was rated in MA mode, again meaning those more independent in ambulation (higher FAC), rated walking in in MA mode as less natural. Overall, these trends point to more dependent individuals rating walking in the device as more natural. In contrast, a moderate positive relationship was found between FAC and comfort in AA mode, with a corresponding negative relationship for MRS, pointing to more physically independent and ambulatory participants rating the device as more comfortable when they could contribute to the movement.

**Figure 1:**
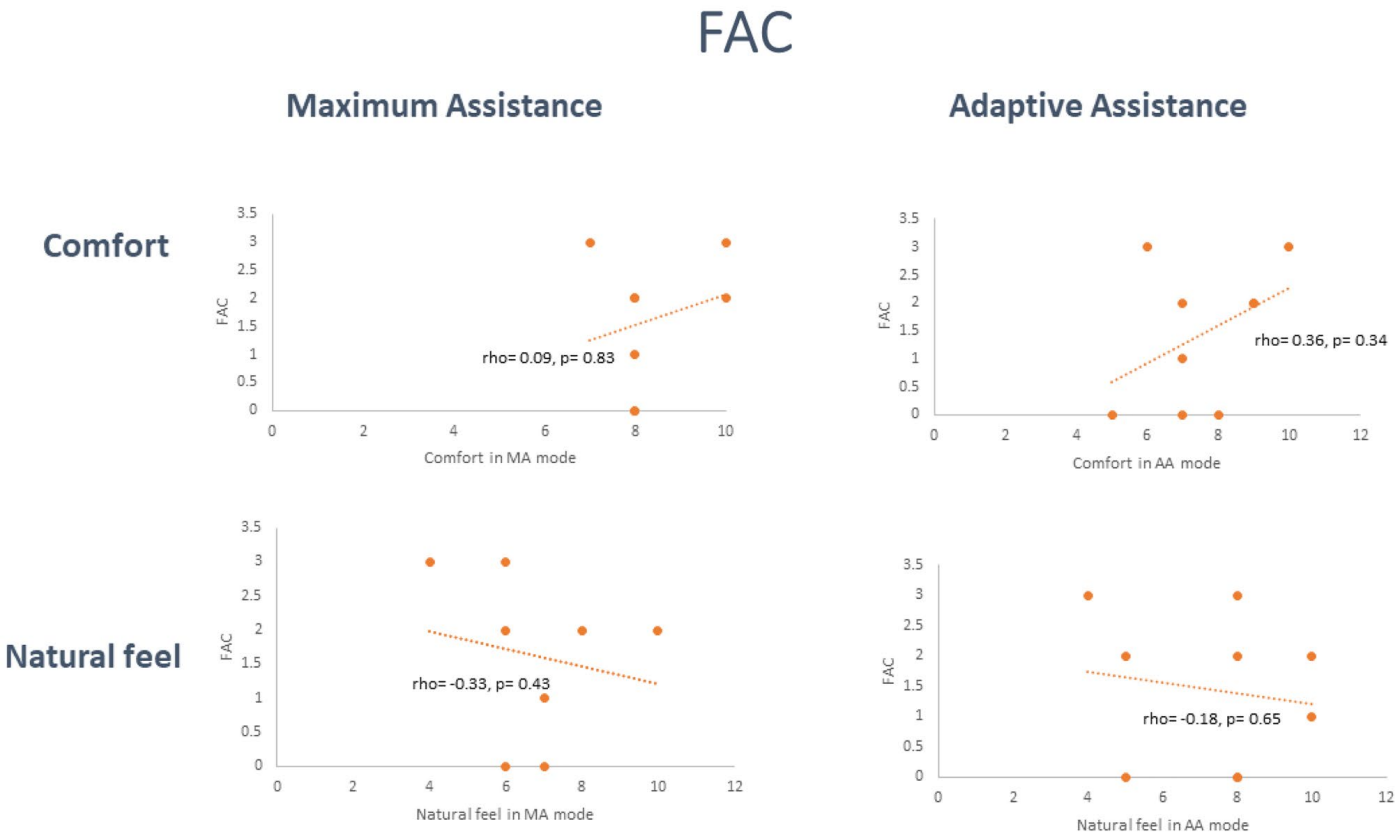
Correlation between FAC and Likert rating scales.

**Figure 2:**
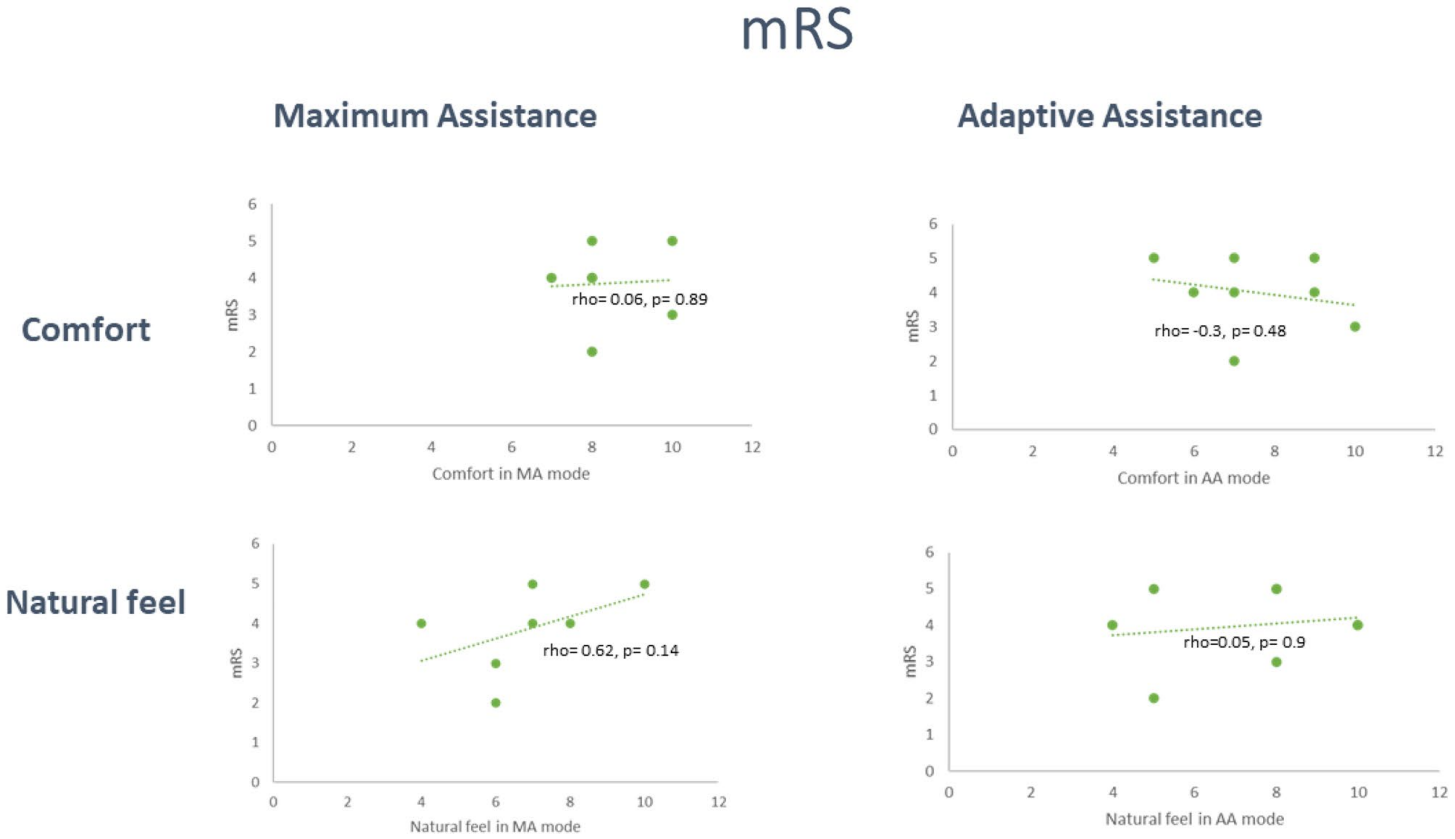
Correlation between mRS and Likert rating scales.

Results from Spearman’s correlation coefficient of the relationship between ambulatory ability (FAC) and disability (mRS) and Likert scales in AA and MA mode are depicted graphically in Figs. 1 and 2 respectively.

## Discussion

This study provided new insights into robotic-assisted gait devices when utilised in the acute hospital setting from individuals in the acute and subacute phases of stroke. The results suggest individuals in the early phases of stroke perceive a robotic-assisted gait device in an acute setting to be acceptable and helpful in the main. Participants identified set-up time, weight of the device, and requirement of multiple instructions by therapists during walking as negative aspects of the device. The majority of participants believed that the device could provide gait training benefits and would choose to continue using the device. On average the device was rated as comfortable to use and the robotic-assisted walking in both maximum and adaptive assistance modes were rated as natural.

Qualitative meta-synthesis of robotics in rehabilitation identifies that robotic therapy is perceived as enjoyable, but also tiring, frustrating and difficult, with quantitative data supporting the enjoyment and fatiguing elements^24,25^. These sentiments were mirrored to a large degree in our research in the acute hospital setting after stroke where participants were positive about their experiences of RAGT but with caveats that related to the weight and restrictive nature of the device and the need for repeated therapist verbal instructions during use. Despite these, the majority of participants in the current study considered RAGT as useful and beneficial and would choose to add RAGT to their rehabilitation programme, if given the choice. In stroke research, comparative qualitative data relating to RAGT are limited and conflicting. A study by Vaughan-Graham et al.^22^ reported that chronic stroke survivors while hopeful about exoskeleton technology, expressed disappointment that the technology did not yet meet their expectations^22^. In contrast more recent qualitative and quantitative studies conducted in subacute stroke populations report more positive perceptions or ratings of exoskeleton-based gait training which better echoing the findings in this study, where patients rated it favourably, attributing opportunity and benefit to using the device during subacute rehabilitation^23–25^.

Time demands for device set-up was one limitation highlighted in the current study that may potentially impede widespread uptake among acute and subacute stroke survivors within a clinical facility. The amount of time required to record individual anthropometric measurements and fit the device were identified as negative aspects. This limitation was mirrored in other qualitative studies examining device users'^21–23^ and therapists’ perspectives^23,34–36^ of RAGT. However, this current study recorded only early perceptions following each participant's first two trials of using the device where the burden of assessment and setup has previously been reported as highest^35^, and findings need to be carefully interpreted in this context. Similarly, while individuals in the current study identified the technology was daunting at first, studies with longer training periods have identified a phase described by patients as “getting into the swing of things”^23^. Qualitative data addressing both user and therapists’ perceptions of the device previously highlighted the importance of committing to a minimum number of training sessions when integrating an exoskeleton into stroke rehabilitation, and that perseverance through early difficulties is necessary to attain efficient exoskeleton training^23^. This finding is further supported by a feasibility study that examined the integration of RAGT in inpatient rehabilitation, where therapists initially reported that time limitations reduced the effectiveness of the device but reported an improvement in feasibility over time^34^.

This study adds to the existing knowledge base of patient experiences using RAGT following stroke by providing quantitative ratings relating to walking in a robotic device in both maximum and adaptive assistive modes. Overall, the device was rated as comfortable to use and the robotic-assisted walking in both modes was rated as natural. This is consistent with the existing evidence where maximal assistance mode was previously rated positively for device comfort by participants in the subacute phase of stroke^24,25^. Data analysis in the current study generated some interesting findings. While all participants rated the device as relatively comfortable (> 5 on the Likert scale), the MA mode (where the person is a passive passenger in the device) was perceived to be more comfortable than the AA mode (where the person contributes to the movement). One reason participants may have perceived MA mode to be more comfortable is that they are not attempting a movement that may be at odds with the programmed trajectory. RAGT in healthy individuals has previously been noted to limit lower limb and pelvic degrees of freedom resulting in altered muscle activation patterns^37^. A second reason for this finding may be as a result of the increased energy demands of walking in AA mode when compared to MA mode. While there is no comparable evidence published in stroke, a study in incomplete spinal cord injury found a wide variation in cardiorespiratory and metabolic responses based on the modes of assistance used, with the maximum assistance mode reported to be the least demanding on cardiorespiratory and metabolic functions^38^. The level of assistance provided to participants by the device in adaptive assistance mode was not recorded nor the variation in assistance between limbs and this must be considered as a limitation in interpreting the findings fully.

All participants in this study again gave a relatively positive rating for how natural walking in the device felt (> 5 on the Likert scale). Here, in contrast to the findings in relation to comfort, ratings were higher in the AA mode, where the individual contributes to the stepping trajectory. We hypothesise that it feels more natural for participants to initiate walking themselves and to have more autonomy over the movement than to be a passive passenger in the device. While no data were identified in the published literature for direct comparison between robotic modes of assistance, a previous study reported individuals with spinal cord injury reported walking felt more natural when they had the autonomy to trigger a step forward using forward and lateral weight shift targets in comparison to when it was triggered by a therapist, even if they were not always in tune with the device^39^. It is interesting to note that in the current study that a strong linear relationship was found between disability level (measured by the mRS) and the natural feel of the device in MA mode, where a higher level of disability related to a more natural perception of walking in the device in MA mode. This supports the interpretation that an individual's perception of how natural robotic walking feels is dependent on their level of impairment and their current compensatory techniques during normal overground walking. Current robotic exoskeleton devices may promote more unnatural, non-physiological walking characteristics to those of an independent ambulator’s natural gait. Evidence supports that walking in a robotic gait device (such as EKSO) is dissimilar to normal overground walking with regard to lower extremity muscle activity and joint motions. In healthy individuals this manifests as slower walking, with shorter steps and greater double-limb support time when using the device^40,41^. However, EMG and EEG studies in robotic-assisted gait identify greater movement intent and muscle contribution in active rather than passive walking in robotic-assisted gait devices^42^ and it is this active contribution to gait that may be driving the higher ratings for natural gait in active assistance mode.

## Conclusion

Overall exoskeleton gait training was deemed to be an acceptable intervention that was comfortable, natural and helpful in gait rehabilitation for individuals with stroke in the acute hospital setting. Participants identified that the technology could be daunting at first and that people using the device should keep an open mind, relax, and trust the device and therapists. Some negative aspects identified included device set-up time, the weight of the device, and the need to respond to multiple verbal commands when walking in the device.

## Supplementary Information

Supplementary Information.

## Data Availability

The data that support the findings of this study are not openly available due to reasons of sensitivity and are available from the corresponding author upon reasonable request.

## Abbreviations

RAGT: Robotic Assisted Gait Training
MA mode: Maximum Assistance mode
AA mode: Adaptive Assistance mode
FAC: Functional Ambulation Category
mRS: modified Rankin Scale
