# Supplementary material for: Stroke survivor perceptions of using an exoskeleton during acute gait rehabilitation
Source: Sci Rep. 2022 Aug 19;12:14185. doi: 10.1038/s41598-022-18188-7 (PMC9391354; doi:10.1038/s41598-022-18188-7)
Supplement: Supplementary file 1 — Supplementary Information. [file 41598_2022_18188_MOESM1_ESM.docx]

Table 1: Detailed participant profile

| **ID** | **Age**  **(years)** | **Sex** | **Height (m)** | **Weight (kg)** | **Time since stroke (days)** | **Stroke Type** | **Stroke laterality (Right/ Left)** | **Stroke location Artery/ Territory** | **mRS pre-stroke** | **mRS post-stroke** | **mAS–** | **Cognitive/ consciousness screening (MoCA*/ OCS**/ CRS-R***)** | **Communication difficulty** | **FAC pre-stroke** | **FAC post-stroke** | **Level of assistance required for ambulation** | **Type of assistive devices or orthotic used** |
| --- | --- | --- | --- | --- | --- | --- | --- | --- | --- | --- | --- | --- | --- | --- | --- | --- | --- |
| 01 | 76 | F | 1.63m | 67.6kg | 19 | Ischemic | Left | PCA | 0 | 4 | Hip flexors: Left 0 / Right 0  Hip extensors: Left 0 / Right 0  Knee flexors:  Left 0 / Right 0  Knee extensors: Left 0 / Right 0  Ankle plantar flexors:  Left 0 / Right 0  Ankle dorsiflexors: Left 0 / Right 0 | Mild cognitive impairment  (MoCA 21/30) | Nil difficulties | 5 | 2 | Ax1  (Ambulant) | Rollator frame |
| 02 | 90 | F | 1.52m | 37.4kg | 28 | Ischemic | Left | PCA | 4 | 5 | Hip flexors: Left 0/ Right 0  Hip extensors: Left 0 / Right 0  Knee flexors:  Left 0 / Right 0  Knee extensors: Left 0 / Right 0  Ankle plantar flexors:  Left 0 / Right 0  Ankle dorsiflexors: Left 0 / Right 0 | Moderate cognitive impairment  (MoCA 10/30) | Mild to moderate hypokinetic dysarthria. Reduction in intelligibility of speech. | NR | 2 | Ax1  (Ambulant) | None |
| 03 | 67 | F | 1.73m | 92.4kg | 14 | Ischemic | Left | MCA | 1 | 2 | Hip flexors: Left 0 / Right 0  Hip extensors: Left 0 / Right 0  Knee flexors:  Left 0 / Right 0  Knee extensors: Left 0 / Right 0  Ankle plantar flexors:  Left 0 / Right 0  Ankle dorsiflexors: Left 0 / Right 0 | OCS areas of impairment:  Sentence reading: 14/15  Broken hearts test:48/50  Delayed recall:  -A. Verbal memory; free recall: 0/4  -B. Verbal memory; Recognition: 3/4  -C. Episodic Memory; Recognition: 4/4. | Expressive difficulties | NR | 2 | Ax1  (Ambulant) | None |
| 04 | 61 | M | 1.67m | 56kg | 42 | Ischemic/ haemorrhagic | Left | MCA | NR | NR | Hip flexors: Left 0 / Right 0  Hip extensors: Left 0 / Right 0  Knee flexors:  Left 0 / Right 0  Knee extensors: Left 0 / Right 0  Ankle plantar flexors:  Left 0 / Right 0  Ankle dorsiflexors: Left 0 / Right 0 | OCS areas of impairment:  Picture naming: 3/4  Sentence reading: 10/15  Meaningless gesture imitation: 11/12  Delayed recall:  A- Verbal memory: Free recall: 1/4  B- Verbal memory: Recognition: 4/4  C- Episodic memory: Recognition: 4/4  Executive Task: 11 | Expressive aphasia,  English second language – translator required | NR | 0 | Ax2  (Non-ambulant) | Hoist |
| 05 | 47 | F | 1.6m | 62.6kg | 50 | Ischemic | Right | MCA | 0 | 4 | Hip flexors: Left 0 / Right 0  Hip extensors: Left 0 / Right 0  Knee flexors:  Left 0 / Right 0  Knee extensors: Left 0 / Right 0  Ankle plantar flexors:  Left 1 / Right 0  Ankle dorsiflexors:  Left 0 / Right 0 | Mild cognitive impairment  (MoCA - 23/30) | Expressive and receptive language intact at basic level.  Following 2 and 3 stage commands (higher level not assessed in MMUH). | 5 | 3 | Ax1  (Ambulant) | None |
| 06 | 61 | M | 1.68m | 65kg | 51 | Ischemic | Left | ACA, MCA | 0 | 4 | Hip flexors: Left 0 / Right 0  Hip extensors: Left 0 / Right 0  Knee flexors:  Left 0 / Right 0  Knee extensors: Left 0 / Right 0  Ankle plantar flexors:  Left 0 / Right 0  Ankle dorsiflexors: Left 0 / Right 0 | OCS completed.  Areas of impairment:  Sentence reading: unable to complete due to language barrier.  Meaningless gesture imitation: 11/12  Delayed recall:  -A. Verbal memory; free recall: 0/4 – as above, unable to complete.  -B. Verbal memory; Recognition: 0/4 as above, unable to complete.  -C. Episodic Memory; Recognition: 4/4 Executive Task: 3 | Moderate-severe non-fluent expressive aphasia - Stronger receptive language skills: able to read at single word level and understand single stage commands.  English second language – translator required | 5 | 1 | Ax2  (Step transfer, otherwise non-ambulant) | None |
| 07 | 63 | M | 1.78m | 71.4kg | 79 | Ischemic | Left and Right | L-ICA, L-M1, R-M1 | 0 | 5 | Hip flexors: Left 0 / Right 0  Hip extensors: Left 0 / Right 0  Knee flexors:  Left 0 / Right 1  Knee extensors:  Left 0 / Right 0  Ankle plantar flexors:  Left 0 / Right 0  Ankle dorsiflexors: Left 0 / Right 0 | OCS completed (Ax limited due to patients’ communication impairment- only areas 1-3 assessed)  Areas of impairment: Semantics: 0/3  Orientation: 2/4 | -Severe motor speech deficits.  -Stronger receptive skills: can indicate yes/no in response to direct  Questions. | 5 | 0 | Ax2  (Non-ambulant) | Hoist |
| 08 | 60 | M | 1.7m | 58kg | 57 | Ischemic | Left and Right | Basilar vessel occlusion | 0 | 5 | Hip flexors: Left 0 / Right 0  Hip extensors: Left 0 / Right 0  Knee flexors:  Left 0 / Right 0  Knee extensors: Left 0 / Right 0  Ankle plantar flexors:  Left 0 / Right 0  Ankle dorsiflexors: Left 0 / Right 0 | CRS-R 15/23  Strong level of conscious awareness. Follows basic commands | Severe dysarthraphonia. Consistently follows verbal information | 5 | 0 | Ax2  (Non-ambulant) | Hoist |
| 09 | 73 | M | 1.78m | 94.5kg | 10 | Ischemic | Left | PCA | 0 | 3 | Hip flexors: Left 0 / Right 0  Hip extensors: Left 0 / Right 0  Knee flexors:  Left 0 / Right 0  Knee extensors: Left 0 / Right 0  Ankle plantar flexors:  Left 0 / Right 0  Ankle dorsiflexors: Left 0 / Right 0 | Mild cognitive impairment  (MoCA 20/30) | Nil difficulties | 5 | 3 | Supervision  (Ambulant) | None |
| 10 | 47 | M | 1.8m | 63.5kg | 39 | haemorrhagic | Right | Frontal lobe | 0 | 5 | Hip flexors: Left 0 / Right 0  Hip extensors: Left 0 / Right 0  Knee flexors:  Left 2 / Right 0  Knee extensors: Left 0 / Right 0  Ankle plantar flexors:  Left 0 / Right 0  Ankle dorsiflexors: Left 0 / Right 0 | Mild cognitive impairment  (MoCA 24/30) | Significant cognitive communication impairment.  Fluent verbal output is verbose and tangential. | 5 | 0 | Ax2  (Step transfer,  otherwise non-ambulant) | None |

(F: Female, M: Male, NR: Not Recorded, PCA: Posterior Cerebral Artery, MCA: Middle Cerebral Artery, ACA: Anterior Cerebral Artery, ICA: Internal Carotid Artery, mRS: modified Rankin Scale, MMUH: Mater Misericordiae University Hospital, FAC: Functional Ambulation Category, mAS: modified Ashworth Scale, MoCA: Montreal Cognitive Assessment, OCS: Oxford Cognitive Screen, CRS-R: Coma recovery scale- revised, Ax: Assessment, Ax1: Assistance of one individual, Ax2 Assistance of two individuals)

*MoCA score classification: 18-25 = mild cognitive impairment, 10-17= moderate cognitive impairment, and less than 10= severe cognitive impairment.

**OCS score classification: Areas where cognitive impairment was identified are described. Areas where participants scored full marks are detailed and should be considered without impairment. A score of one or more points above or below the normative value, where relevant, is considered a deficit in that area of cognition.

***CRS-R score classification: A score of 8 or higher indicates a ‘strong level of conscious awareness’.
